# Muscular dystrophy in the *mdx* mouse is a severe myopathy compounded by hypotrophy, hypertrophy and hyperplasia

**DOI:** 10.1186/s13395-015-0041-y

**Published:** 2015-05-01

**Authors:** William Duddy, Stephanie Duguez, Helen Johnston, Tatiana V Cohen, Aditi Phadke, Heather Gordish-Dressman, Kanneboyina Nagaraju, Viola Gnocchi, SiewHui Low, Terence Partridge

**Affiliations:** Center for Genetic Medicine Research, Children’s National Medical Center, 111 Michigan Avenue NW, Washington DC, 20010 USA; Myology Center of Research, Institut de Myologie Pitié-Salpétrière - Bâtiment Babinski, 75651 Paris Cedex 13, France; Center for Genetic Muscle Disorders, Kennedy Krieger Institute, 801 N. Broadway, Baltimore, MD 21205 USA; Department of Embryology, Carnegie Institution for Science, Baltimore, MD 21218 USA

**Keywords:** Muscular dystrophy, Muscle regeneration, Hypertrophy, Hypotrophy, Hyperplasia

## Abstract

**Background:**

Preclinical testing of potential therapies for Duchenne muscular dystrophy (DMD) is conducted predominantly of the *mdx* mouse. But lack of a detailed quantitative description of the pathology of this animal limits our ability to evaluate the effectiveness of putative therapies or their relevance to DMD.

**Methods:**

Accordingly, we have measured the main cellular components of muscle growth and regeneration over the period of postnatal growth and early pathology in *mdx* and wild-type (WT) mice; phalloidin binding is used as a measure of fibre size, myonuclear counts and BrdU labelling as records of myogenic activity.

**Results:**

We confirm a two-phase postnatal growth pattern in WT muscle: first, increase in myonuclear number over weeks 1 to 3, then expansion of myonuclear domain. *Mdx* muscle growth lags behind that of WT prior to overt signs of pathology. Fibres are smaller, with fewer myonuclei and smaller myonuclear domains. Moreover, satellite cells are more readily detached from *mdx* than WT muscle fibres. At 3 weeks, *mdx* muscles enter a phase of florid myonecrosis, accompanied by concurrent regeneration of an intensity that results in complete replacement of pre-existing muscle over the succeeding 3 to 4 weeks.

Both WT and *mdx* muscles attain maximum size by 12 to 14 weeks, *mdx* muscle fibres being up to 50% larger than those of WT as they become increasingly branched. *Mdx* muscle fibres also become hypernucleated, containing twice as many myonuclei per sarcoplasmic volume, as those of WT, the excess corresponding to the number of centrally placed myonuclei.

**Conclusions:**

The best-known consequence of lack of dystrophin that is common to DMD and the *mdx* mouse is the conspicuous necrosis and regeneration of muscle fibres. We present protocols for measuring this in terms both of loss of muscle nuclei previously labelled with BrdU and of the intensity of myonuclear labelling with BrdU administered during the regeneration period. Both measurements can be used to assess the efficacy of putative antinecrotic agents. We also show that lack of dystrophin is associated with a number of previously unsuspected abnormalities of muscle fibre structure and function that do not appear to be directly associated with myonecrosis.

## Background

From shortly after its discovery in the early 1980s [1], the *mdx* mouse fell into disrepute and was widely dismissed as a model of human muscular dystrophy [2-5]; that its muscles are hypertrophic and of similar absolute strength to those of the C57Bl/10 wild-type (WT) mouse did not fit well with the muscle-wasting phenotype of Duchenne muscular dystrophy (DMD) boys. Its eventual revelation as a genetic homologue of DMD [6] subsequently established it as the most-used preclinical model of DMD but with the common proviso that the *mdx* pathology is much less severe. This comparison is based mainly on the progression of muscle fibrosis and, importantly, of clinical incapacity expressed as a proportion of the lifespans of man and mouse, a debatable basis for comparison of pathological severity between two such different species [7]. Persistence of such opinions is abetted by a lack of well-authenticated quantitative criteria in either species by which such comparisons might validly be made. While current technologies permit us to gather detailed quantitative information on gene expression and proteomic profiles in DMD and *mdx* dystrophies [8-10], at the level of cellular pathology, we lack equivalent data to permit accurate translation of molecular events into pathological processes.

To realize the full potential utility and to accommodate the limitations of the *mdx* mouse as a preclinical model requires a deeper understanding of its pathology by comparison with that of DMD in man. A full quantitative description of the processes involved in generating pathological changes over the course of disease, especially of the dynamics of those processes, could provide a basis for determining the role of each pathological feature of the *mdx* dystrophy in the development of the disease. Such information is required to fully evaluate the applicability, or otherwise, to DMD of data emerging from this animal model and to better interpret results from tests of potentially therapeutic preclinical interventions.

To understand those features that are common to the two species and those that differ between them, we need, at a minimum, to develop quantitative methods in the mouse for measuring and comparing the benefits to pathology and function of any putative therapeutic treatment. At present, we have only a hazy understanding of the relationship between the pathological targets that we are aiming to modify with a given treatment and the outcome criteria that are commonly assessed in such investigations. For example, variation in fibre size, the frequency of centrally placed myonuclei or the deposition of fibrous connective tissue are all recognized as consequent to dystrophic pathological processes in the *mdx* mouse, but their relationships to the primary pathology unleashed by the lack of dystrophin are indirect and obscure. Likewise, although the molecular mechanisms by which putative treatments are thought to modify these processes are often identified, the cellular mechanisms that translate these molecular changes into structural and functional improvements remain largely unresolved. As a first approach to gaining a better understanding of these various relationships, we have made a detailed examination of the *mdx* mouse muscle during its phase of postnatal growth, driven by conspicuous satellite cell activity, and of its transition, at the end of this period, into a florid myopathic stage that is accompanied by even more intense satellite cell activity.

We have assessed these events by three main methods. First, we have monitored the accumulation of myonuclei within muscle fibres as a measure of the contribution of myogenic cell fusion to fibre growth. Second, the content of fibrous actin has been measured as an index of the amount of contractile material within each muscle fibre. Third, we have followed the incorporation of the thymidine analogue BrdU into muscle fibre nuclei as a measure of myogenic activity and loss of these labelled nuclei as an indicator of myonuclear turnover in the period of growth and at the onset of myopathology in *mdx* mice.

We demonstrate a number of previously unrecognized consequences of absence of dystrophin from skeletal muscle. In particular, muscle growth in *mdx* mice is markedly compromised prior to the onset of overt myonecrosis; growth of *mdx* fibres lags behind that of WT fibres, with fewer myonuclei that have smaller sarcoplasmic domains. This hypotrophy is accompanied by feeble attachment of satellite cells to the *mdx* fibres but appears to originate pre- or peri-natally. At 3 weeks, WT muscle growth involving satellite cell activity ceases, further enlargement being accomplished by expansion of the myonuclear domain. In contrast, *mdx* mice at this age enter a sudden myonecrotic phase and myogenic activity becomes yet more conspicuous, coping with both the hypertrophic growth and the repair of extensively destroyed muscle fibres. At maturity, *mdx* muscles and their component myofibres are larger than those of WT and are hypernuclear, with more than double the number of myonuclei per fibre. Thus, although hypertrophic in terms of absolute bulk, muscles of the *mdx* mouse can be considered hypotrophic in terms of myonuclear domain, which falls to half the WT value. We propose that the *mdx* mouse differs greatly from DMD in having no problem of repair and regeneration. This brings into question its value as a model of this aspect of dystrophinopathy. However, the basic similarity of myonecrotic events commends it as a model for testing therapies aimed at modifying this aspect of DMD.

## Methods

### Animals and BrdU and EdU administration

C57Bl10/ScSn mice and *mdx* C57Bl10/ScSn mice were managed and handled according to protocols approved ethically and scientifically by the local animal care and use committee guidelines of the Children’s National Medical Center, Washington, DC. For some experiments, mice were injected subcutaneously with BrdU (30 mg/kg) morning and evening for the first postnatal week. Administration of BrdU to post-weaning mice was by inclusion in their drinking water (0.8 mg/ml) for a period of 1 week. In some experiments, pre-weaning mice were injected twice daily with EdU, which is more easily visualized but too expensive to be administered in the drinking water and is more toxic than BrdU in longer term administration protocols.

Mice aged 1, 2, 3, 4, 6, 14 and 28 weeks were euthanased by CO_2_ flooding followed by cervical dislocation, and tibialis anterior (TA), gastrocnemius and extensor digitorum longus (EDL) muscles were dissected from both hindlimbs.

### Isolation of myofibres

Reagents were from Invitrogen (Thermo Fisher Scientific, Grand Island, NY, USA) unless otherwise stated. Single myofibres were isolated as described previously [11]. Briefly, right limb EDL muscles were carefully dissected immediately after euthanasia of the mouse and incubated in 0.2% Collagenase Type 1 (Sigma-Aldrich, St. Louis, MO, USA) in DMEM for 1 to 2 h, depending on muscle size and age, to digest the connective tissue. Single myofibres were liberated by gentle trituration with fire-smoothed wide-mouthed Pasteur pipettes in DMEM in dishes pre-coated with horse serum. Liberated myofibres were washed by transfer through four such dishes. TA and gastrocnemius muscles of mice that had been given BrdU were frozen on corks in isopentane held at freezing point in liquid nitrogen for subsequent cryostat sectioning.

### Immunostaining

EDL muscles from 16-day-old *mdx* and WT mice (*n* = 4) were frozen in liquid-nitrogen-cooled isopentane. Transverse sections cut at −20°C were incubated for 1 h at RT in permeabilization/blocking buffer (TBS-T, 0.5% Triton X-100, 2% BSA 2% (*w*:*v*), 20% goat serum) and then overnight at RT in primary antibodies diluted in permeabilization and blocking buffer. Anti-laminin-2 (clone 4H8-2, 1:400, Axxora, Lausen, Switzerland) was used to define fibre margins and LAMP1 (dilution 1:300, H-228, Santa Cruz Biotechnology, Santa Cruz, CA, USA) to visualize lysosomal structures. Slides were then washed three times in TBS-T 10 min at RT prior to incubation in secondary antibodies (goat anti-rat Alexa Fluor 488, 1:400, Invitrogen; donkey anti-rabbit Alexa Fluor 595, 1:400, Invitrogen) diluted in permeabilization and blocking buffer. After three 10-min washes in TBS-T at RT, the slides were counter-stained with DAPI, 1 μg/ml for 2 min, rinsed two times and mounted with Gel Mount (Sigma). Four images of 1-μm-thick optical sections were gathered from each section, using Zeiss LSM 510 Meta NLO on an Axiovert 200 M microscope (Carl Zeiss, Jena, Germany). Images were acquired using AxioVision software (Carl Zeiss).

BrdU staining with biotin-conjugated anti-BrdU (Life Technologies B35138) 1:100 overnight, followed by Alexa 488 streptavidin (Invitrogen S32354) 1:500, was performed according to the supplier’s instructions. Laminin co-staining was performed by overnight incubation at 4°C with anti-laminin antibody (Sigma L9393) 1:400, followed by Marina Blue goat-anti-rabbit IgG (Invitrogen M10992) 1:500 for 1 h at RT to reveal the primary antibody localization.

EdU-treated mice were euthanased on postnatal day 18 and EDL muscles were harvested. Single myofibres were isolated as described above. EdU detection was performed on fixed myofibres using the Click-iT Assay kit (Life Technologies, Carlsbad, CA, USA) according to the manufacturer’s instructions. DAPI was used to visualize nuclei.

### Isolation, immunostaining and counting of Pax7-positive cells

Myofibres were isolated as described above, fixed with 4% paraformaldehyde and immunostained with anti-Pax7 antibody (Developmental Studies Hybridoma Bank, Iowa City, IA, USA) as previously described [12].

### Fixation and phalloidin staining of myofibres

Batches of myofibres harvested at each age were transferred to a 2-ml Eppendorf vial containing 1.5 ml 3.65% formaldehyde and fixed at 37°C for 15 min. The vial was centrifuged at 700 *g* for 10 min, to loosely pellet the myofibres. The formaldehyde supernatant was carefully removed, and 1 ml of 30% sucrose was added to the pellet which was resuspended by a 3-s vortex and stored at −80°C pending analysis.

For staining, the vials were thawed at room temperature and the contents deposited into a Petri dish together with the products of a rinse with TBS-Tween (Tween 0.1% in TBS). Using a stereo microscope and watchmaker’s forceps, myofibres were carefully transferred into a 2-ml Eppendorf vial containing permeabilization and blocking buffer (0.5% Triton, 0.1% Tween, 2% BSA and 20% goat serum) in which they were incubated overnight at 4°C. After centrifugation for 10 min at 15,000 *g*, the buffer was removed and the fibres were rinsed three times with alternate centrifugations in 1 ml of rinse solution (0.1% Tween in TBS).

The myofibres were then re-suspended in Alexa Fluor 594-conjugated phalloidin at a dilution of 1:40 for 20 min at room temperature, as previously described [11], rinsed three times and left in the rinse solution overnight at 4°C to maximize elution of unbound phalloidin. After centrifugation for 10 min at 15,000 *g*, myofibres were re-suspended in a 0.75 μg/ml solution of DAPI and immediately centrifuged for a further 10 min at 15,000 *g*, then rinsed once more. The contents of the vial were deposited into a Petri dish and transferred gently, using a stereo microscope and forceps, and mounted onto a microscope slide.

### Imaging and software analysis

Single fibre imaging and quantification of F-actin was performed as previously described [11]. Briefly, stained myofibres were imaged on a Nikon Eclipse E500 epifluorescence microscope with Spot Camera (Nikon Corporation, Chiyoda-ku, Japan). Overlapping segments of each myofibre were recorded under a Nikon PlanApo × 10 objective. Between two to six images were required, depending on the length of the myofibre. Exposure time was set to fall within a previously validated linear response range of signal intensities [11] and was kept constant across all samples within a comparison. Images of each myofibre were stitched together using the CS2 version ‘Interactive Layout’ mode of the Photomerge feature of Adobe Photoshop in Adobe Photoshop Elements.

For measurement of volume, confocal microscopy z-stacks were acquired on a Zeiss LSM 510 Meta NLO system with an Axiovert 200 M microscope. Depths ranged from 35.3 to 61.8 μm, depending on myofibre thickness. Slices of 2.2 μm thick composed of 512 × 512 pixels were collected with × 20 objective. Between two to six non-adjacent z-stacks were collected per myofibre, depending on myofibre length. DAPI-stained nuclei were counted for each z-stack by eye from three-dimensional representation using AxioVision software (Zeiss) and added together. Volume data of each segment are expressed per myonucleus within that segment.

Integrated density of signal from fluorophore-conjugated phalloidin for each slice was calculated in ImageJ and added together to give total signal per z-stack. Signals per z-stack were added for each myofibre and divided by the number of nuclei to give signal per nucleus.

### Measurement of growth of muscle fibres

To follow growth of muscle fibres from WT and *mdx* mice, we recorded the two main contributory factors: the numbers of nuclei within the muscle fibre (myonuclei) and the amount of filamentous actin (F-actin). Since most fibrous actin within the fibre is associated with the myofibrillar structures, this is an index of the amount of contractile apparatus. These methods were developed and analysed for consistency and reproducibility in a previous study [11]. EDL muscles were dissected from WT and *mdx* mice aged from 1 to 28 weeks of age (three animals per time point) and dissociated in type 1 collagenase as described above. Batches of fibres obtained from *mdx* and WT mice were thawed in age-matched pairs, subjected to the staining regime, and mounted for microscopic examination.

### Validation of fluorescent phalloidin signal as an index of volume

**Figure 1:**
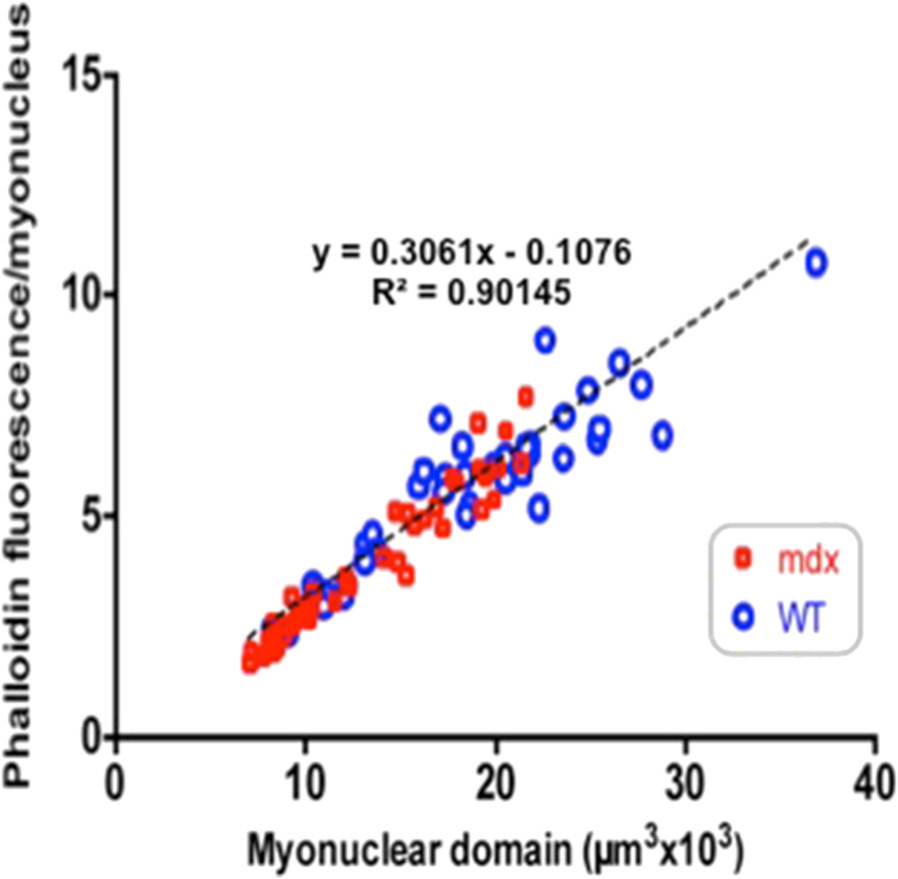
Plot of fluorescent phalloidin signal per myonucleus against estimates of fibre volume per myonucleus from stacked confocal images visualized in segments of muscle fibres isolated from *mdx* and WT muscles at ages of 2, 6 and 28 weeks. The strong linear relationship between the two parameters across the age range and the two mouse strains validates the use of phalloidin fluorescence as a reliable index of fibre volume within these limits.

To determine the relationship between fibrous actin content and sarcoplasmic volume, we compared the phalloidin signal from segments of individual muscle fibres extracted from EDL muscle fibres of 2-, 6- and 28-week *mdx* and WT mice with the calculated volumes of the same segments from z-stacks of confocal images as described previously [11]. These two measures were normalized to the number of myonuclei in these same segments and plotted against one another (Figure 1). The strong correlation between these two parameters across the two strains and spanning the entire age range validates the use of the fluorescent phalloidin signal as a reliable index of sarcoplasmic volume within these bounds, irrespective of age or strain. Thus, we apply the term ‘myonuclear domain’ as a descriptor of both the amount of contractile material associated with each myonucleus as measured by the fluorescent phalloidin signal and the volume of sarcoplasm calculated from the regression relationship.

### Statistics

For myofibres processed by the PhAct method on a standard fluorescent microscope, statistical tests for significant differences between WT and *mdx* myofibres in terms of nuclear counts (myonuclei, peripheral, centralized or Pax7-positive) and actin signal (per fibre or normalized to the number of myonuclei) were analysed within an ANOVA framework. Some parameters were transformed to achieve normal distributions (square-root transformation: Pax7-positive nuclei per total nuclei and actin signal; log transformation: actin signal per myonucleus, total nuclei and myonuclei), then two-way ANOVA was performed for each age. Factors included into the model were the murine strain and the experimental batch of the myofibres.

For myofibres imaged using confocal microscopy, for which volume per nucleus and actin per volume were measured, because of the non-normality of dependent variables, non-parametric tests were used to compare medians. For the analysis at each time point, the Wilcoxon rank-sum test was used to compare strains.

## Results

### Postnatal fibre growth differs substantially between *mdx* and WT muscle fibres

**Figure 2:**
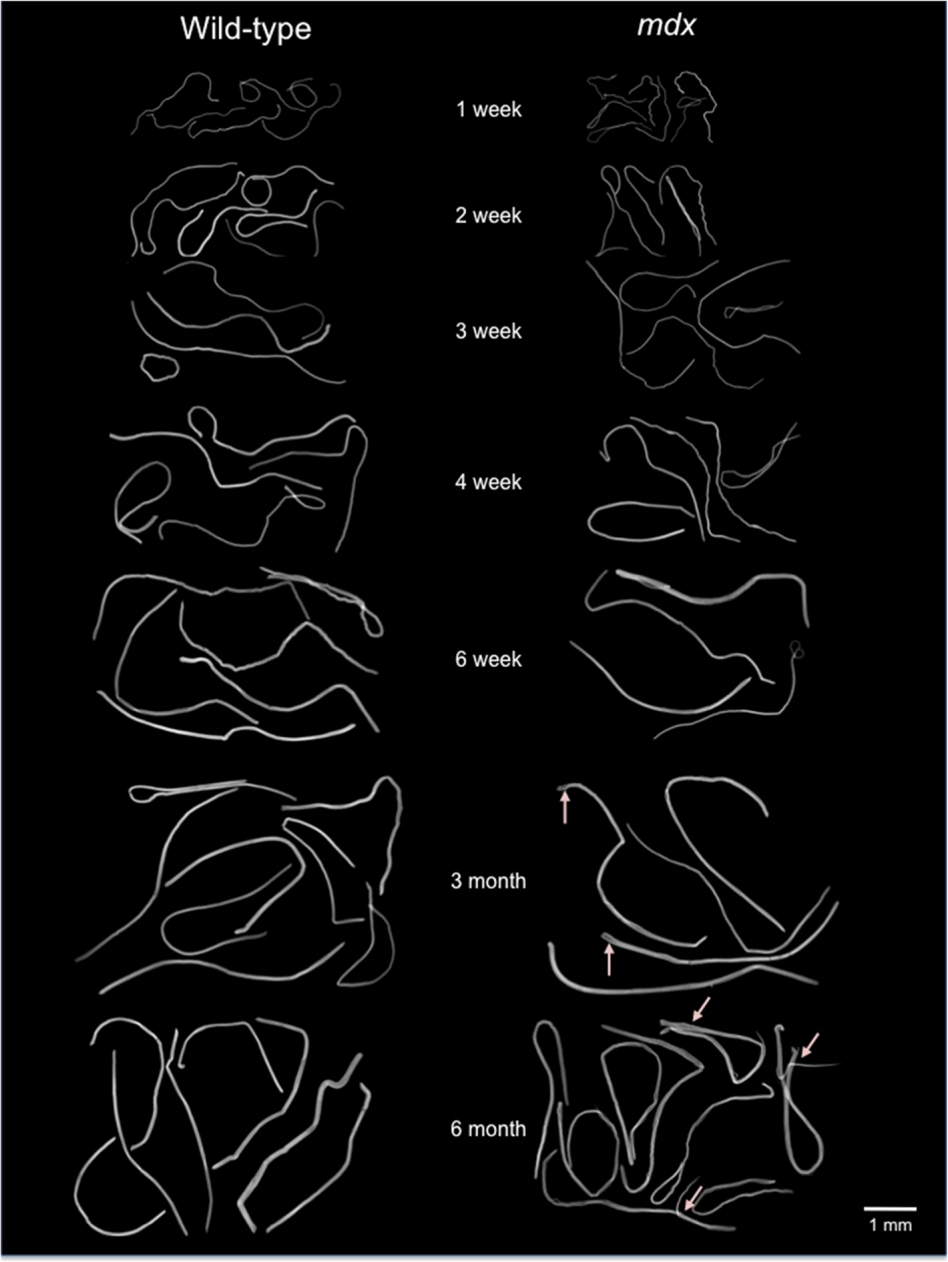
Individual fibres isolated from muscles of *mdx* and WT mice aged from 1 to 28 weeks. The visual impression that muscle fibres of *mdx* mice are thinner than those of age-matched WT controls is borne out by analyses shown in subsequent figures. Branching is evident in the older *mdx* fibres.

**Figure 3:**
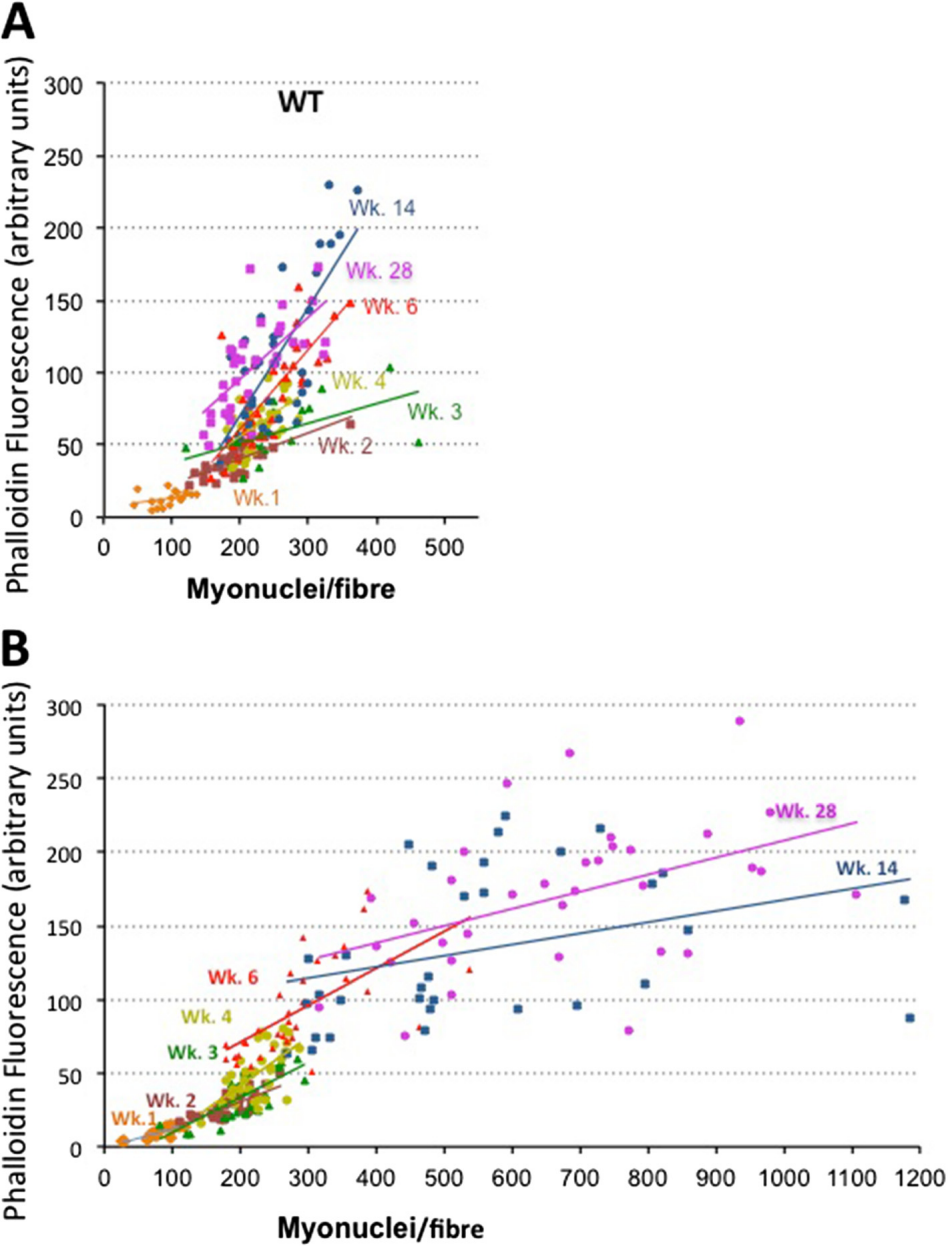
Growth of *mdx* and WT muscle fibres up to 28 weeks of age, showing the total phalloidin fluorescence per myofibre, as an indicator of fibre volume, plotted against the number of myonuclei in that myofibre, displayed as individual data points and linear best fits across the range of ages as identified by colour. **(A)** Growth of WT fibres. Myonuclear number, plotted along the *X*-axis, increases up to week 3 but not thereafter, all further growth being accomplished by increase in fibrous actin content, along the *Y*-axis, as indicated too by the increasing slope of the best fit lines. **(B)** The equivalent plot to **(A)** of *mdx* muscle fibres using the same scales, to show the marked difference between growth patterns of the two strains. Up to week 4, *mdx* growth is broadly similar to that of WT but, beyond this point, continues both by a great increase in number of myonuclei per fibre and by increase in F-actin content. The total F-actin per fibre becomes larger than that of WT, in association with fibre branching, but the ratio of fibrous actin per myonucleus is low, as indicated by the shallow slopes of the best fit lines.

Simple visual comparison of age-matched isolated fibre preparations from WT and *mdx* muscles indicated that for the first 4 weeks, *mdx* fibres tended to be smaller but, beyond 6 weeks, appeared larger than those of WT and showed branching that was not observed in those of WT (Figure 2). To quantify myofibre growth, we analysed myofibres isolated from WT and *mdx* mice by two main criteria: the number of myonuclei and the amount of fibrous actin they contained as plotted in Figure 3A,B. These data confirmed the visual impression that *mdx* myofibres lagged behind those of WT in both respects for the first 4 postnatal weeks but overtook them in terms of absolute size (phalloidin fluorescent yield) in mature animals.

### Myofibre growth in WT mice

As expected from previous studies [13], myonuclear number per WT fibre increased steadily over the first 3 weeks, when it ceased abruptly (Figure 3A). All further growth occurred by increase in the amount of F-actin per fibre, as indicated by the progressive rise along the *Y*-axis of the individual data points in Figure 3A, without further increase along the *X*-axis. The increasing slope of the correlation lines within the WT fibre population also indicates an increase in the F-actin (that is, sarcoplasmic volume) per myonucleus up to 14 weeks of age, with no further increase by 28 weeks.

### Growing *mdx* muscle is hypotrophic prior to the onset of disease

For the first 3 to 4 weeks, muscle fibres of *mdx* mice followed a largely similar pattern of increase in myonuclear number per fibre accompanied by a small increase in fibrous actin content (Figure 3B). But, thereafter, the myonuclear number per *mdx* fibre increased progressively along the *X*-axis to more than twice that seen in WT muscles of equivalent age. The F-actin content also increased to higher average levels than that of WT but were highly variable and not in proportion to myonuclear number.

**Figure 4:**
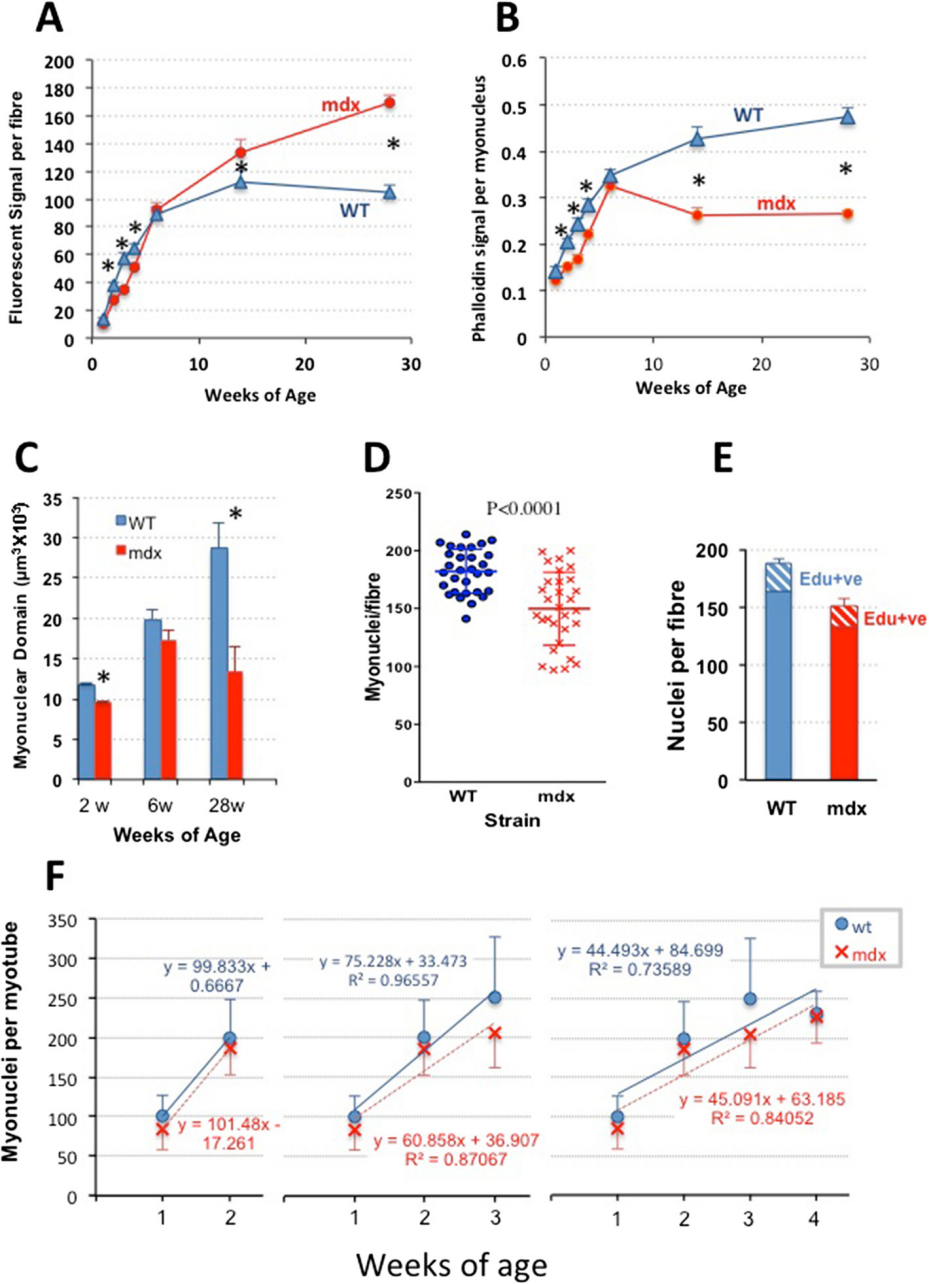
Difference between patterns of myogenesis in WT and *mdx* mice. **(A)** Fluorescent phalloidin signal per myofibre (mean ± SEM) plotted against age, illustrating the lag in *mdx* fibre growth in up to 6 weeks but progressive hypertrophy beyond this point (*P < 0.05). **(B)** The fluorescent phalloidin signal per nucleus (mean ± SEM) also increases rapidly in both mouse strains up to 6 weeks, but with *mdx* lagging significantly (*P < 0.05) behind WT. The subsequent continuing rise in WT fibres contrasts with the fall to half of the WT values in *mdx*. **(C)** Column plot of the estimated myonuclear domains in muscle fibres isolated from muscles of 2-, 6- and 28-week WT and *mdx* mice (mean ± SEM, *P < 0.05). **(D)** Scatterplots, together with depiction of mean ± SD, of myonuclear number per myofibre isolated from 18-day-old WT and *mdx* mouse EDL muscles. **(E)** EDL fibres from mice labelled with EdU at 14, 15 and 16 days of age and analysed on day 18 showing similar labelling frequencies of fibre-associated nuclei (new-formed myonuclei plus Pax7-positive satellite cells). **(F)** Nuclear number/fibre (mean ± SD) of fibres isolated from muscles of *mdx* and WT mice at weeks 1 to 4. The successive incremental plots show a progressive fall in slope from 100 nuclei/fibre between weeks 1 and 2 to a net rise of approximately 45 nuclei per fibre averaged across weeks 1 to 4. Cessation of WT growth is evident beyond week 3. Myonuclei numbers in *mdx* fibres fall consistently below those of WT fibres, but the plots run largely parallel suggesting that the intensity of myogenesis in the two strains is closely similar and that the *mdx* fibres had fallen behind WT during prenatal development.

The difference between patterns of myogenesis in WT and *mdx* mice is depicted more succinctly as plots of the average fluorescent phalloidin signal per muscle fibre (Figure 4A) and per myonucleus (Figure 4B) across the range of ages.

In WT myofibres, the fluorescent signals per myofibre and per myonucleus both increase continuously up to 14 weeks of age, where they stabilize.

In *mdx* myofibres, by contrast, both relationships are biphasic, following two distinct courses. Up to the onset of frank myonecrotic disease at 3 to 4 weeks, the fluorescent phalloidin signal, both per myofibre and per myonucleus, lags significantly behind that of the age-matched WT. This confirms the visual impression in Figure 2 that individual *mdx* fibres are smaller in these young mice (Figure 4A). Calculation of the myonuclear domain from the fluorescent phalloidin yield (Figure 4C) confirms that this too is significantly smaller in *mdx* than in WT fibres in these young growing muscles as well as later in the adult mice.

### Hypotrophic growing *mdx* muscle fibres contain fewer myonuclei than WT fibres

Up to week 4, fewer myonuclei were routinely seen in *mdx* than in the age-matched WT myofibres but not significantly so at any given time point. We therefore conducted a larger experiment to determine whether the difference was real and whether myogenic activity was lower in *mdx* mice during this postnatal growth period. Three WT and three *mdx* mice were each injected twice per day with EdU (0.5 mg/g) from p13 to p15, prior to euthanasia at 18 days. Analysis of ten isolated EDL fibres per mouse showed that *mdx* myofibres contained significantly fewer myonuclei than age-matched WT fibres (Figure 4D). However, the proportion marked by a 3-day administration of EdU over this period, which would comprise both myogenic cells in mitosis over the labelling period and myonuclei derived from them, did not differ significantly between WT and *mdx* (Figure 4E).

We conclude that for the first 3 to 4 postnatal weeks, *mdx* muscle fibres are hypotrophic, in terms of all parameters we have measured; that is, they are of significantly smaller volume and contain significantly fewer myonuclei with significantly smaller sarcoplasmic domains than the age-matched WT fibres.

Detailed plots of the myonuclear numbers in fibres over weeks 1 to 4 (Figure 4F) confirm that the lower myonuclear numbers in *mdx* myofibres are not attributable to differences in rate of proliferation of myogenic cells. They show that the accumulation of myonuclei in *mdx* and WT fibres rose in parallel over this growth period but with the *mdx* lagging slightly behind the WT; both showed an increase of 100 myonuclei per fibre between weeks 1 and 2, dropping to a mean increase of 60/75 myonuclei per fibre by week 3 and further, to approximately 45 when averaged over the first 4 weeks. We conclude that lack of myonuclei over the postnatal growth period does not reflect a lower rate of myogenic cell proliferation and fusion during this time and that it must have arisen at or before birth.

In the context of continuously degenerating/regenerating muscle beyond week 3, the low F-actin content per myonucleus is unsurprising [14] but the seeming lag in growth of this factor prior to this time was unexpected. The small myonuclear domain in these young *mdx* is not attributable, as it is during the subsequent myopathic stage, to excessive myonuclear numbers and must therefore reflect a diminished maintenance of sarcoplasmic protein per myonucleus during this growth phase.

**Figure 5:**
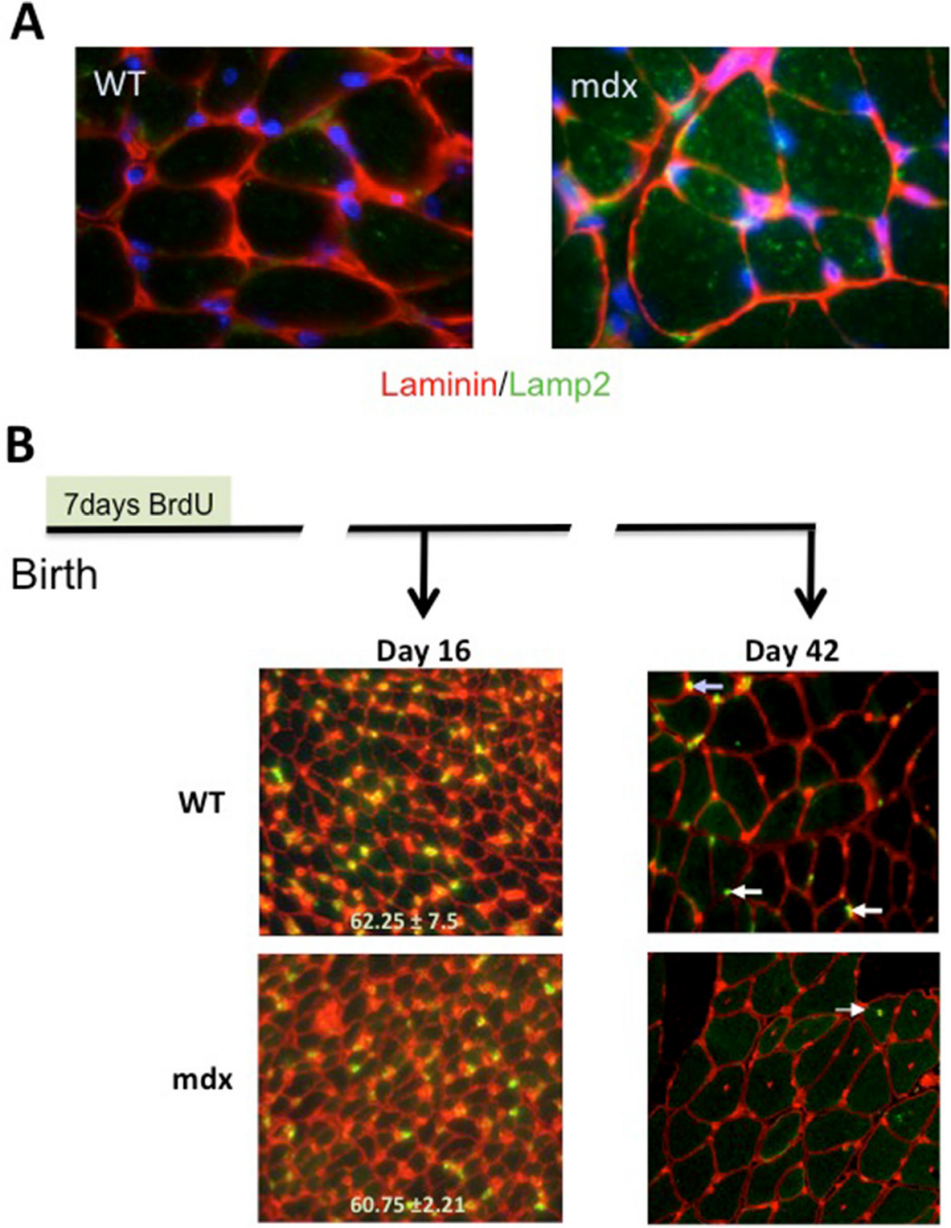
*Mdx* muscles show excess lysosomal activity prior to disease and massive turnover after onset. **(A)** Cryostat sections of TA muscles from 2-week-old *mdx* and WT muscles immunostained for laminin (red) to show fibre outlines and Lamp1 (green) and DAPI (blue) to show the strong representation of lysosomes within *mdx* fibres as a possible contributory factor to the hypotrophic condition of these fibres. **(B)** Immunostains for BrdU (green) and laminin (red) counter-stained with propidium iodide (red) of the section of TA muscles from mice injected twice daily with BrdU during the first week of life and euthanased at 16 or 42 days of age. At 16 days, prior to the onset of myonecrosis, muscle from both strains showed similar frequencies of labelled nuclei. *N* = 4 muscles. Data are shown as mean ± SD. In the WT muscle, the BrdU label had been retained at 42-day muscle, both by identifiable myonuclei (arrowed) and by interstitial cells but had been lost, apart from the occasional rare (1 to 2 per whole section) centrally placed labelled myonucleus in the *mdx* muscles.

Thus, the postnatal hypotrophy of *mdx* EDL muscle arises from two factors: a smaller myonuclear domain in combination with fewer myonuclei per fibre than WT. In previous tissue culture studies, we had observed a similar hypotrophic pattern to that seen here *in vivo*, that is, hyponucleated myofibres in the context of equivalent overall myogenicity and small myonuclear domain; this in association with elevated lysosomal content as well as with excess secretion of protein in lysosomal vesicles [15]. As shown in Figure 5A, the hypotrophic *mdx* fibres in pre-necrotic *mdx* muscle *in vivo* are also heavily loaded with lysosomes, suggesting that similar aberrations of protein handling and secretion may contribute to the hypotrophic status of these fibres.

### Onset of myopathy in *mdx* mice involves a switch from hypotrophy to a hyperplasia-driven hypertrophy

Beyond 3 to 4 weeks of age, the phalloidin signal per *mdx* myofibre increases more rapidly than WT with age. This reflects an increase to a very large size of mature *mdx* myofibres (Figures 3B and 4A) accompanied, at 14 and 28 weeks, by an even greater increase in myonuclear number with the result that sarcoplasmic volume per myonucleus, that is the myonuclear domain, was reduced to half of that of WT fibres at these ages (Figure 4B,C).

### Onset of *mdx* myopathy involves massive turnover of muscle fibres

To examine the dynamics of the initial growth and transition from growth into disease, we injected a cohort each of newborn *mdx* and WT mice with BrdU subcutaneously twice per day (morning and evening) on postnatal days 1 to 7. At 16 days of age, counts of labelled muscle fibre nuclei per unit area of muscle from *mdx* and WT mice (Figure 5B, *n* = 4 mice) showed no difference between the two strains, adding to the preceding evidence that the lower myonuclear content in *mdx* compared to that in WT muscle fibres over the first 3 weeks is not attributable to diminished myoblast proliferation during this period.

By 42 days of age, muscle sections from four mice from each of these same labelled cohorts showed a stark divergence between the two strains in retention of labelled nuclei. In WT muscles, numerous BrdU-labelled nuclei were identified both in myonuclear positions within the myofibres and in the interstitium between fibres. By contrast, in *mdx* muscles at this age, only a very rare isolated labelled nucleus per whole muscle section was found in either position. Since myonuclei do not divide, loss of labelled myonuclei from *mdx* muscle must reflect loss of the nuclei themselves, presumably associated with death and replacement of the fibre, rather than by the main alternative, namely, mitotic dilution of the BrdU label to subliminal levels. For the interstitial nuclei, either explanation is possible. Overall, the loss of BrdU-stained myonuclei indicates that the majority of muscle fibres had been lost and replaced over the 3 weeks between the onset of the myopathy and time of examination. Thus, muscle precursor cell activity over this 3- to 6-week period had not only replaced the complete muscle mass but, as shown explicitly in Figure 3B and by implication from the halving of the myonuclear domain in Figure 4B,C, had augmented the total myonuclear content by approximately 100% over the number in the WT mouse.

### The changing relationship between satellite cells and muscle fibres during postnatal growth and regeneration in *mdx* muscles

**Figure 6:**
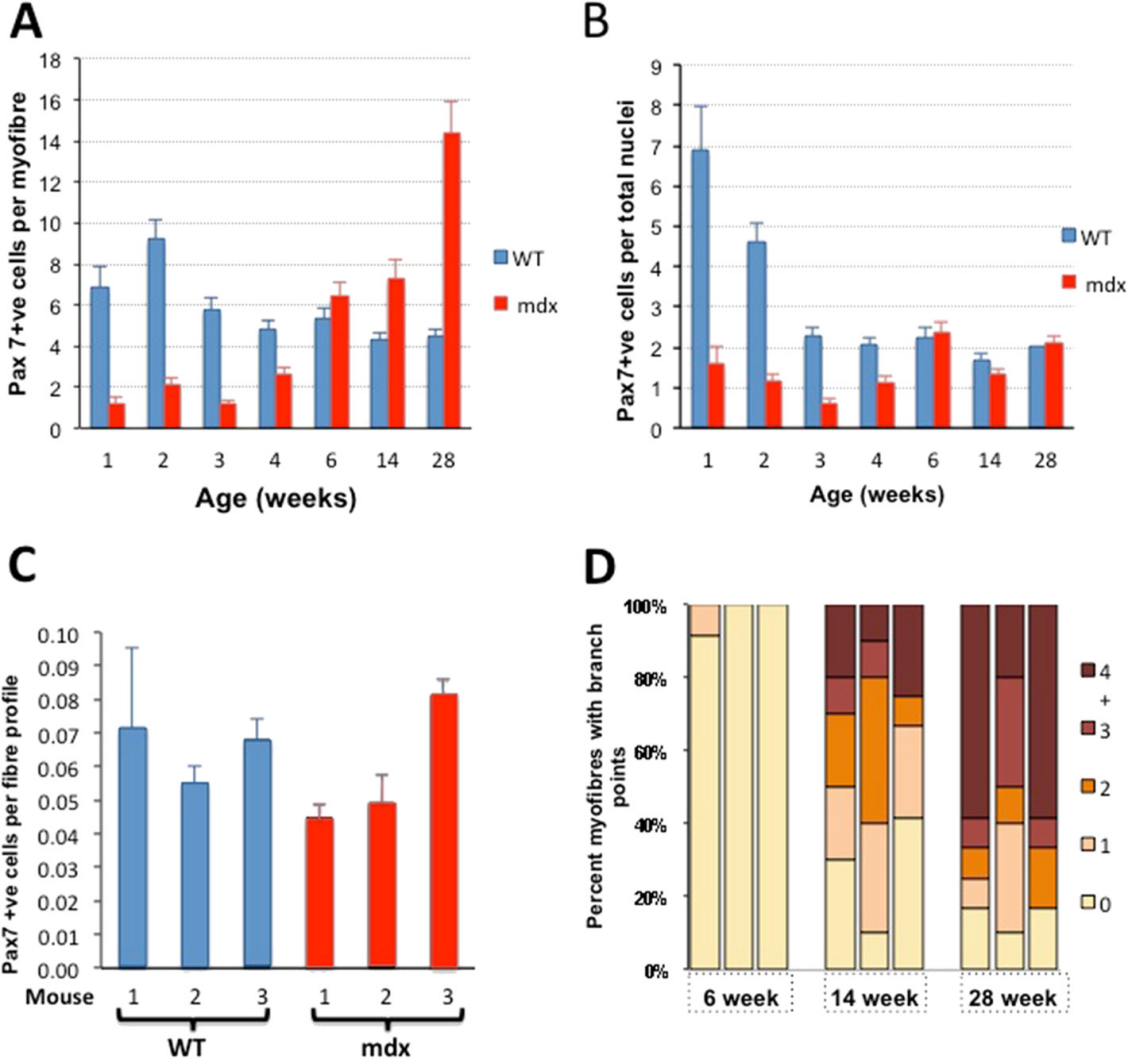
Numbers of Pax7-positive satellite cells and of fibre branches in *mdx* and WT muscles. **(A)** Numbers of satellite cells on myofibres extracted from EDL muscles of 1- to 28-week-old WT and *mdx* mice, shown as Pax7-positive cells per fibre (mean ± SEM). Up to week 4, *mdx* fibres carried markedly fewer satellite cells than those of WT. Beyond 6 weeks of age, the numbers of satellite cells on *mdx* fibres increased progressively and significantly exceeded the WT number at 14 and 28 weeks.**(B)** Pax7-positive cells on fibres extracted from EDL muscles of 1- to 28-week-old WT and *mdx* mice, normalized to total myofibre nuclei (mean ± SEM). Again, during the first 4 weeks, the percentage of satellite cells on *mdx* fibres is markedly lower than that on WT fibres but, at 6 weeks and beyond, the numbers are closely similar, suggesting a link between myonuclear density and satellite cell number in mature muscle. **(C)** Numbers of Pax7-positive cells per myofibre profile in frozen sections of muscle from 2-week-old *mdx* and WT mice (three individual mice of each strain). In the sectioned muscle, Pax7-positive cells are equally frequent in WT and *mdx* mice at this age. **(D)** Frequency of fibres bearing one to four branches in muscles of *mdx* mice at 6, 14 and 28 weeks of age. No branches were seen on fibres extracted from three batches of *mdx* mice younger than 6 weeks or from WT mice at any age examined, but the frequency and complexity of branching increased progressively with age of the *mdx* mouse.

Counts of Pax7-positive satellite cells on WT fibres over the first 3 weeks corresponded well to those found by White et al. [13], but muscle fibres isolated from *mdx* muscles carried far fewer of these cells than their age-matched WT equivalents (Figure 6A,B). This reconciles poorly with the similar rates of incorporation of new myonuclei into growing fibres of the two strains over this period (Figure 4F) or the similar frequencies of incorporation of EdU and BrdU labels into nuclei during postnatal weeks 1 to 3 (Figures 4E and 5B). Suspecting that satellite cells were being lost preferentially from these young *mdx* muscle fibres during the isolation process, we counted Pax7-positive cells, per fibre profile, in cryostat sections of EDL muscles at 14 days of age and found no significant difference between *mdx* and WT muscles (Figure 6C). Thus, Pax7-positive cells were present with equal frequency *in situ* in these young *mdx* and WT EDL muscles but were lost preferentially from *mdx* fibres during the isolation procedure, suggesting that their adhesiveness to these young growing fibres was reduced in the absence of dystrophin.

By 6 weeks of age, *mdx* muscle fibres carried equivalent numbers of satellite cells to those of WT muscle fibres and the frequencies of satellite cells per *mdx* fibre rose consistently over the remaining period of observation to higher numbers than those of satellite cells per WT fibre (Figure 6A). Interestingly, the satellite cell number standardized to myonuclear number per fibre was constant over the entire period from 6 to 28 weeks (Figure 6B). Maintenance of so similar a proportionality in such dissimilar conditions as the WT and *mdx* muscles implies some hitherto unremarked interaction between the myonuclear population and satellite cell number.

### Fibre splitting increases progressively with age in *mdx* muscle

**Figure 7:**
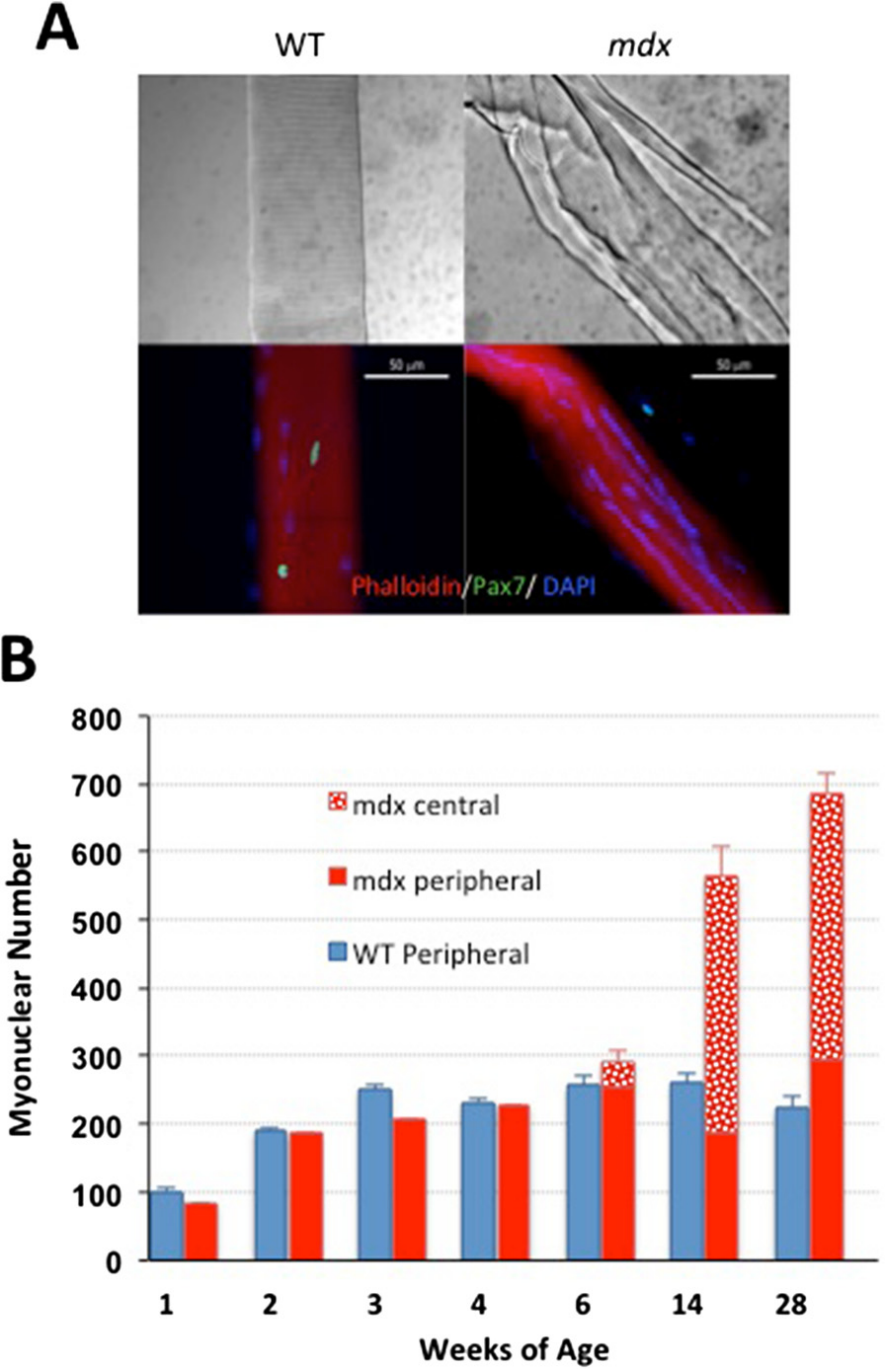
Increase with age in the proportion of ‘central nuclei’ and of fibre branching in *mdx* mice. **(A)** Interference contrast and fluorescent micrographs of single fibres isolated from 1-year-old *mdx* and WT mice and immunostained for Pax7. These not only bear readily identifiable satellite cells (green) but also illustrate the extensive branching of fibres in older *mdx* but not in WT mice together with the tendency for myonuclei to be arranged in linear ‘central strings’. **(B)** Counts of myonuclei classified into peripheral location and ‘central’ location, the latter being classified on the basis of their distribution in linear arrays. These ‘central’ myonuclei are first seen in 6-week-old *mdx* mice. At 14 and 28 weeks, however, it is notable that the numbers of peripherally located myonuclei per fibre are not substantially different from those of the WT and that the centrally located nuclei account for all of the excess myonuclei found in older *mdx* muscles.

Beyond 6 weeks of age, we show a significant increase in *mdx* myofibre volume, as indicated by fluorescent phalloidin signal, that is not proportionate to the more than doubling of myonuclei. The reported hypertrophy of *mdx* mouse muscles is not matched by increased fibre diameter [16] but, as shown in Figures 2, 6D and 7A, is accompanied by a progressive increase in the frequency of branched or split fibres [17-19], which has recently been shown to play a major role in *mdx* muscle hypertrophy [19].

### ‘Central’ nuclei account for the myonuclear excess in *mdx* over WT

A noted feature of *mdx* mouse muscle and of regenerated muscle in the adult mouse in general [20] is the large proportion of nuclei distributed in centrally placed strings [20,21] (Figure 7A). In our counts, we designate myonuclei in aligned strings as ‘central nuclei’ (that is, not in the normal juxta-sarcolemmal position) in distinction from those not in this configuration which we classify as peripheral myonuclei. In this investigation, we find no central strings of nuclei in the WT fibres at any age. In *mdx* fibres, they are first seen at 6 weeks of age and increase rapidly by 14 and 28 weeks. Counts reveal that these ‘central’ myonuclei account fully for the higher total myonuclear number in *mdx* muscle fibres (Figure 7B). In fact, the numbers of myonuclei located peripherally in the regenerated myofibres remain constant beyond 4 weeks of age in *mdx* mouse muscle and do not differ significantly from the numbers of total myonuclei per fibre in age-matched WT muscles. Although the supernumerary myonuclei in older *mdx* muscle correspond numerically to the central nuclei, myonuclei in both positions are marked by 1 week of BrdU labelling and we are unable, at present, to identify either with any specific aspect of the regenerative process.

### Phasing of muscle growth and regeneration is not limited to the EDL

**Figure 8:**
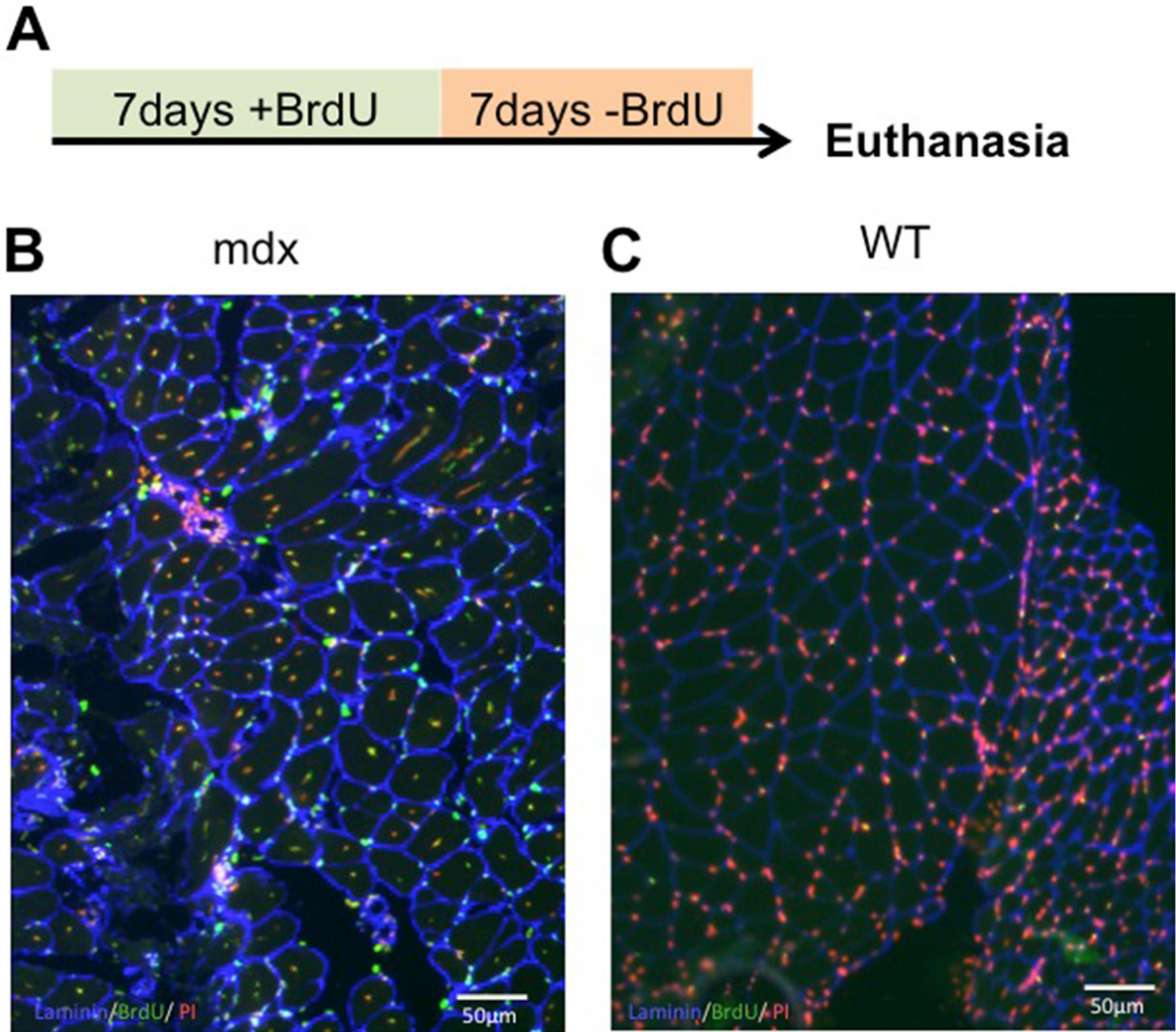
Assessment of myogenic activity in muscles of *mdx* and WT mice. **(A)** Protocol for labelling cell participation in muscle regeneration. Mice are given drinking water containing 0.8 mg/ml BrdU for 7 days and maintained on normal drinking water for a further 7 days before being euthanased for analysis. **(B)** Section from a gastrocnemius muscle taken from a *mdx* mouse subjected to the regime in **(A)**. Seven days after the end of BrdU administration, it shows extensive labelling of both interstitial cells and centrally located myonuclei in a spontaneous *mdx* muscle lesion (green or yellow). Virtually all of the centrally placed myonuclei are labelled, showing that the BrdU had not been diluted to subliminal levels by proliferation of the myogenic cells that had repaired this particular lesion. **(C)** Gastrocnemius of a WT mouse subjected to this same labelling regimen. An occasional interstitial nucleus is stained, but there are no centrally placed myonuclei and no identifiable myonucleus is stained. Thus, the cessation of myonuclear-based growth at 3 weeks, identified in the isolated fibres of the WT EDL muscles, is a generalized phenomenon extending to the gastrocnemius muscle and to other limb muscles examined.

Our picture of a cessation of satellite cell-based growth of WT muscle at 3 weeks of age is based on investigation of fibres from the EDL muscle. To determine whether this was more broadly true, we gave BrdU to mice in their drinking water from weeks 4 to 5, looking for signs of continuing proliferation of myogenic cells in a range of muscles. As outlined in Figure 8A, mice were euthanased after a further week on water not laced with BrdU to give any myogenic cells labelled during week 4 ample time to differentiate and enter a growing or regenerating muscle fibre. Gastrocnemius and TA muscles of *mdx* mice subjected to this protocol showed conspicuous labelling of a significant proportion of myonuclei within focal areas of regeneration, in which virtually all myonuclei were labelled with BrdU (Figure 8B), reflecting synchrony of regeneration within such spontaneous lesions and implying that the proliferation of myogenic cells had not diluted the label to subliminal levels within this protocol. Muscles of WT mice subjected to this same regimen showed only occasional BrdU labelling of interstitial cells and no labelling of myonuclei (Figure 8C).

## Discussion

It is of practical concern that, although the *mdx* mouse has been the animal model of first resort for preclinical testing of potential therapeutic ideas and agents for treatment of DMD, we lack a coherent pathological picture of it. The common assertion that the *mdx* dystrophy is milder than DMD remains unsupported by quantitative data in either species [7]. Here, we have gathered detailed quantitative information outlining the main features that distinguish the *mdx* mouse from the WT mouse during its postnatal growth and the transition to severe pathology. Although comparison with DMD is constrained by the scanty human data, we do identify several similarities and differences between *mdx* and WT that would invite further investigation in man as well as highlighting a number of features of dystrophinopathy that are not apparent from more casual observation of either species.

Of practical interest, we delineate a set of readily measurable criteria for use in assessment of the beneficial effects of putative therapeutic strategies. We also identify several features that make this animal less than ideal, sometimes misleading, as a model of DMD. Recognition of such issues is important if we are to avoid developing therapeutic strategies based on misconception.

### Hypotrophy in growing *mdx* muscles

This is the first report of a lag in growth of *mdx* EDL muscle fibres behind that of WT during the early postnatal growth period. It is a hypotrophy; small fibre size arises from a combination of fewer myonuclei, each with a smaller myonuclear domain. We dismiss the idea that lower myonuclear numbers in isolated *mdx* fibres during this period are attributable to the low numbers of satellite cells, since this is revealed as an artefact by the comparable satellite cell frequency at 2 weeks in histological sections of *mdx* and WT muscles (Figure 6C). This bespeaks excess satellite cell loss during fibre isolation from young *mdx* compared with WT mice, implying a looser association of satellite cells with *mdx* than with WT fibres that might itself predispose to slower growth. But other evidence argues against this. Even at week 1, *mdx* fibres contained fewer myonuclei than WT fibres, while the actual rate of growth across the 3 weeks was indistinguishable between the two (Figure 4F), as were the rates of incorporation of EdU (Figure 4E) and BrdU (Figure 5B). These data converge on the idea that the difference had arisen at or before birth and merely persisted over the postnatal myogenic growth period. A reported paucity of Pax7-positive cells in early development in the *mdx* mouse [22] might be held responsible for this, but the need for sequestration of satellite cells beneath the basement membrane during late prenatal growth [23] provides an alternative mechanism potentially susceptible to disturbance of the dystrophin-dependent link between the basement membrane and the fibre plasmalemma [24]. A hangover of this disturbance would also account for the excessive loss of satellite cells during isolation of fibres from *mdx* muscle during early postnatal growth [25].

The features of pre-myonecrotic *mdx* muscle fibres paint a picture of a more complex role of dystrophin in muscle fibre function than simply sustaining resilience of the surface. Thus, the mechanisms that bind satellite cells tightly to the fibre surface are defective, and the small *mdx* fibre size is associated with a small myonuclear domain. Both accord well with our previous *in vitro* studies where *mdx* myotubes were atrophic, hyponuclear, highly enriched in lysosomal structures and chronically exported large amounts of intact protein in aberrant lysosomal vesicles [15]. We show here that this is reflected *in vivo* by hypotrophy and excess lysosomal content of pre-necrotic muscle fibres (Figure 5A). Taken together, these data portray a broad and pervasive derangement of the metabolic and protein degradation processes, which precede myonecrosis [25-27], as integral features of dystrophinopathy.

### The early phase of the *mdx* mouse myopathy is severe

We show that myonuclei labelled during the first postnatal week are largely lost by 7 weeks, 3 to 4 weeks after the onset of the myopathy (Figure 5B). This indicates massive myonecrosis and regenerative replacement, as echoed in the heavy BrdU labelling of myonuclei during weeks 4 to 5 in newly regenerated foci within all muscles examined (Figure 8B). Thus, the lag in growth in *mdx* muscle up to week 4 is fully compensated by week 6 by active myofibre regeneration (Figure 4A), even in the face of massive contemporaneous myonecrosis; this finding fits badly with the idea of distinct and separate phases of early myonecrosis followed subsequently by repair [28].

Overall, these findings argue against attribution of the mild clinical deficiency in *mdx* mouse to a less aggressive primary pathology than DMD. Indeed, far from being milder than DMD, it is doubtful whether the intensity of myonecrosis seen in 4- to 6-week *mdx* mice would be survivable by a DMD boy. Instead, our data show that the *mdx* mouse endures severe early myonecrotic pathology but is better able than man to cope with this by virtue of a more effective and harmonious regenerative response, as epitomized in Figure 8B. Differences in scale between mouse and man may play some part in this [7], but a more fundamental distinction between the *mdx* and DMD pathologies is the different timing of the phases of growth and regeneration in the two species. Satellite cell-dependent growth in the mouse ceases at 3 weeks, coincident with the onset of myonecrosis and regeneration, with no overlap between the separate programmes followed by satellite cells undertaking these two activities [29]. By contrast, in growing boys, myogenic cell proliferation continues throughout the juvenile and pubertal periods [30,31], presenting every prospect of adverse interference in DMD boys between the separate growth and regeneration programmes. Thus, some benefit might be achieved by blockade of discrepant signals between these two different satellite cell functions.

### Quantitative evaluation of disease severity

One objective of this investigation was to develop robust quantifiable criteria by which to evaluate the severity of the *mdx* myopathy and any benefits of putative therapies. Because the spontaneous *mdx* lesions are focal, episodic and fleeting [32], there is considerable experimental noise between individual small samples such as histological sections, so we have broadened the sample space by adoption of two strategies based on labelling proliferating 1-week cohorts with BrdU.

1. Twice-daily injection of BrdU for the first postnatal week labels a neonatal cohort of muscle nuclei whose loss provides a dramatic signal against which to test any therapeutic agent. However, the intensive administration is difficult to standardize and the rapidity of loss of this labelled cohort limits the period of testing of therapeutic actions to the initial myopathic period.
2. We therefore favour the simpler and readily standardized protocol depicted in Figure 8A, whereby BrdU is added to the drinking water for 1 week to deliver a chronic supply of the label to the cohort of cells proliferating during that week, giving any labelled cells that are destined to participate in myogenesis ample time to do so during the subsequent BrdU-free week. As seen in Figure 8B, virtually every identifiable myonucleus is labelled by BrdU in regions of intense regeneration triggered by the spontaneous focal myopathy. Thus, this protocol fully samples events where myogenic proliferation falls completely within the week of BrdU administration as well as partially sampling those that overlap this week. It also shows that the serial cell divisions within any single regenerative event do not dilute the label to below its subliminal threshold. We can thus measure the number and size of focal lesions occurring within a given week and the intensity of labelling within each lesion. In our hands, this regimen labels myonuclei in the *mdx* mouse up to at least 18 months of age and is currently being used to compare regeneration across the *mdx* mouse lifespan and to investigate the effectiveness of exon-skipping treatments in suppressing the *mdx* myopathy.

### Muscle hypertrophy and hyperplasia of myogenic cells

Muscle hypertrophy is an ill-understood feature of dystrophinopathies [33]. Calf muscle hypertrophy is pathognomonic of DMD, although the gradual replacement of muscle with fibro-fatty tissue converts this to ‘pseudohypertrophy’ in older boys [34]. In the GRMD dog, the gluteus, cranial sartorius and tongue show conspicuous hypertrophy [35,36], and in the *mdx* mouse, many muscles are routinely heavier than the equivalent WT [37]. Here, we make new observations pertinent to the role of myogenic proliferation in the hypertrophy.

It remains an enigma that, despite conspicuous myonecrosis, muscle of the *mdx* actually increases in weight beyond 3 to 4 weeks more rapidly than that of the WT and maintains normal levels of absolute strength [14,38,39]. With the possible exception of the diaphragm, there is little evidence that diminished regeneration plays any significant part in the histopathological or physiological decline of *mdx* muscle. Here, we show that hypertrophic regenerated *mdx* muscle fibres are hypernuclear, with double the number of myonuclei per unit volume so that the adult *mdx* mouse, as a whole, contains more than twice as many myonuclei as the WT mouse. This small myonuclear domain features in the hypotrophy in growing pre-myopathic *mdx* and diminishes further after the onset of myonecrosis and regeneration but is more than counter-balanced by the large numbers of myonuclei per fibre. Thus, the myogenic mechanisms in the *mdx* mouse are fully competent to maintain muscle mass, even in the context of massive persistent myofibre degeneration, and must entail extensive hyperplasia of the myogenic precursor pool. In accord with the finding of intact satellite cell number and function in old *mdx* mice [40], we have observed occasional BrdU labelling of mdx muscle up to 18 months (unpublished data). Reports of diminished satellite function with age in the *mdx* mouse (for example [17]) may be attributable to differences between *in vitro* and *in vivo* functional criteria [41,42]. Such exuberant myogenic activity in *mdx* muscle brings into question the utility of boosting myogenesis in this model [43,44]; it would be superfluous to the pathological and physiological demands on the muscle, which are accommodated adequately by the existing mechanisms.

### Satellite cell numbers are maintained in *mdx* muscle

Beyond the first 4 weeks, satellite cell numbers on WT myofibres remain stable, while, in *mdx* muscle, the number per fibre rises steeply, as has been noted previously [17]. However, if normalized to myonuclear number, the satellite cell population remains stable at the approximately 2% level reported for sectioned mouse hindlimb muscles [45] and indistinguishable between *mdx* and WT muscles beyond the 3-week postnatal growth period. Regulation of satellite cell number has been viewed largely in terms of intrinsic, cell lineage-defined, satellite cell qualities that bias the proportion of symmetric versus asymmetric divisions, as influenced by spatial relationships to the basement membrane [46-50]. Our finding that satellite cell number is strongly related to myonuclear number in both WT and *mdx* muscles, even in the context of the abnormal numbers and spatial distributions of myonuclei seen in adult *mdx* muscle, suggests that feedback from the myonuclear population may also impinge on this important issue.

In DMD, the picture is clouded by uncertainty. Attempts to extract and culture myogenic cells have generally concluded that DMD myoblasts are fewer in number and less myogenic than those from healthy human muscle [51,52] and poor proliferation *in vitro* is widely interpreted as evidence of their failure as a causal feature of the pathogenesis [53-59]. At the same time, counts of satellite cells *in situ* have indicated that they are more frequent in DMD than in normal muscle [60-62]. But our ability to investigate the undoubted *in vivo* regeneration deficit in DMD by comparison with normal human muscle is limited by available technology. The discrepancy between the effective regeneration in the *mdx* mouse and its progressive failure in DMD has prompted the breeding of a telomerase-null mutation onto the *mdx* background, aiming to limit the supply of stem cells [63]. But the more general disruptive influence of telomere abnormalities on gene expression, in addition to their effects on cell proliferation, complicates interpretation of data from this animal.

### Role of fibre branching in *mdx* hypertrophy

Since mean fibre diameter does not differ significantly between *mdx* and WT [16,21,64,65], hypertrophy is commonly assumed to reflect an increase in fibre numbers, although in histological sections, it is difficult to distinguish fibre number from fibre branching [18,19,66] and branching itself has recently been shown to be the main component of *mdx* muscle hypertrophy [19]. Thus, the hypertrophy in the *mdx* mouse involves two separate elements: a large increase in the number of myonuclei per fibre and extensive fibre branching that appears to arise from aberrant patterns of myoblast fusion [18,19,67,68].

Interestingly, branching has also been linked to susceptibility of fibres to work-induced damage [69-71], raising the interesting and unresolved question of why the most active degeneration occurs at a time before branches are detected while older muscles, with extensive fibre branching, show relatively little active pathology [16,21].

### Central nucleation equates with hypernucleation

We were surprised to find that the strings of closely adjacent, non-juxta-sarcolemmal, ‘central nuclei’ effectively accounted for all of the hypernucleation in *mdx* muscle fibres. Although the number of peripheral nuclei per fibre remains constant, the excess being centrally located, BrdU marking shows that myonuclei in both positions are renewed during regeneration. The status of central nuclei, whether as an accommodation to the high rates of protein turnover in dystrophic muscle [15,26] or a fundamental defect in control of myonuclear number and placement, remains to be determined. Are they contributors to the myopathic process or a result of it [72]? In any event, it has implications for gene and exon-skipping therapies that are targeted at myonuclei. If these supernumerary myonuclei are transcriptionally active, they represent extra targets for such therapeutic agents; if not, they may act as counter-productive decoys. In non-rodent species, persistent central nucleation is not a conspicuous feature and hypernucleation has not been specifically investigated in muscular dystrophies.

It has been noted that there is a general inverse relationship between myonuclear domain and force production [73] that is contravened in *mdx* muscle, where a halving of myonuclear domain compared with WT is associated with a low specific force [14] adding to the idea that central positioning may reflect a disturbance of normal nuclear/sarcoplasmic relationships. The question of whether DMD and GRMD muscles have high myonuclear densities and whether the persistent central nucleation seen in adult WT mouse muscle after regeneration [20] is clearly of interest in this regard [74,75].

## Conclusions

### Observations

1. We have analysed the growth, loss and regeneration of skeletal muscle in the *mdx* mouse by comparison with the WT mouse.
2. From birth up to the onset of the myopathy, *mdx* mouse muscle grows at the same rate as WT mouse muscle but lags behind it in absolute size, with fewer myonuclei and smaller myonuclear domains than WT and contain excess lysosomal structures.
3. Satellite cells are less adherent to muscle fibres of the young pre-myopathic *mdx* mouse than those of the age-matched WT.
4. After onset of the myopathy, *mdx* muscles become hypertrophic and hypernucleated but the myonuclear domain shrinks to half of the WT size.
5. During the myopathic phase, *mdx* muscle fibres become increasingly branched and accumulate strings of myonuclei that are centrally positioned within the fibres.
6. These central nuclei account entirely for supernumerary myonuclei in *mdx*; the number of peripheral nuclei per fibre remains constant.
7. The number of satellite cells per fibre increases progressively in the *mdx* mouse, but the number per myonucleus remains constant and does not differ from that of the WT.
8. We describe two BrdU labelling protocols to permit quantitative comparison of muscle turnover in the *mdx* mouse.

### Interpretations

Our data show no compromise of regeneration in limb muscles of the *mdx* mouse. Neither satellite cell number nor proliferative capacity is a limiting factor in the maintenance of muscle size and structure in this animal. We suggest that attention to defects in regenerative mechanisms in the *mdx* dystrophy are, as in aging muscle [42], subject to too strong a focus. This distracts from those aspects of the *mdx* myopathy that do closely simulate DMD pathology, such as defects in myofibre stability, where translation of findings from mouse to man is more likely to be of immediate practical value. A more detailed comparison of the cellular and molecular events that are common to DMD and *mdx* pathologies, or peculiar to one or the other, should also prove rewarding in this respect [7,32].

## Abbreviations

BrdU: bromo-deoxy-uridine
DMD: Duchenne muscular dystrophy
EdU: 5-ethynyl-2′-deoxyuridine
GRMD: the golden retriever muscular dystrophy canine model of Duchenne muscular dystrophy
*mdx*: a mouse on C57Bl/10ScSn background that bears a nonsense mutation in exon 23 of the dystrophin gene and thus a genetic homologue of Duchenne muscular dystrophy
WT: the non-dystrophic C57Bl/10ScSn mouse strain
